# Effect of storage time on the stability of selected blood gases, electrolytes, and metabolite concentrations in dairy cattle whole blood and plasma

**DOI:** 10.3168/jdsc.2025-0908

**Published:** 2025-11-07

**Authors:** Marina Madureira Ferreira, Francisco A. Leal Yepes

**Affiliations:** Department of Population Medicine and Diagnostic Sciences, College of Veterinary Medicine, Cornell University, Ithaca, NY 14853

## Abstract

Blood gases, electrolytes, and metabolites assist clinicians and researchers with disease diagnosis and prognosis, and can inform treatment decisions on farms. Delays between sample collection and processing can lead to significant changes in these parameters, affecting their clinical and biological value. Therefore, the objective of this study was to evaluate the stability of selected blood gas, electrolyte, and metabolite measurements in dairy cattle whole blood samples stored at 4°C, in addition to frozen plasma samples stored at −80°C for different storage periods. Multiple blood samples were collected from Holstein lactating dairy cows (n = 10) and preweaning heifer calves (n = 16) at a commercial dairy farm in New York state at a single time point. Samples were analyzed using a blood gas analyzer for sodium (Na), potassium (K), chloride (Cl), ionized calcium (iCa), ionized magnesium (iMg), pH, bicarbonate (HCO_3_^−^), base excess (BE), anion gap (AnGap), glucose (Glu), lactate (Lac), creatinine (Creat), and BUN. One blood sample was analyzed within 30 min after collection to provide baseline values. The remaining 6 samples were stored at 4°C and analyzed after 8, 12, 24, 48, 72, and 168 h. After baseline analysis, the first sample was used to collect plasma samples. The plasma baseline was immediately analyzed, and the 2 aliquots were stored at −80°C for analysis after 2 and 4 wk. The effect of storage time on blood gases, electrolytes, and metabolites was analyzed separately for whole blood and plasma using linear mixed-effects models for each individual blood parameter. Blood concentrations of iCa, iMg, and Creat remained stable during storage at 4°C for up to 168 h. Measurements of pH, Na, K, Cl, Glu, Lac, BUN, BE, and AnGap changed over time, with greater differences observed after 48 h for Glu, BUN, and BE compared with the baseline. Although statistical differences were observed for pH and Na at 24 h, values remained within the normal range until 48 h. The K and Lac were identified as the least stable parameters and underwent significant changes after 8 h. We conclude that bovine whole blood samples can be analyzed for electrolytes, blood gases, and metabolites within 48 h postcollection without significant clinical changes, if well stored and refrigerated at 4°C, except for K and Lac. Plasma samples, however, provide stable and reliable measurements for K and Lac for up to 4 wk of storage at −80°C; the results agree with whole blood concentrations.

## Highlights

• Blood samples stored at 4°C can be analyzed within 48 hours without clinical changes.
• Calcium, magnesium, chloride, anion gap, and creatinine are stable for up to 7 days.
• Potassium and lactate show greater variability over time in whole blood.
• Potassium and lactate are stable in plasma and show good agreement with whole blood.

## Graphical Abstract

**Figure:**
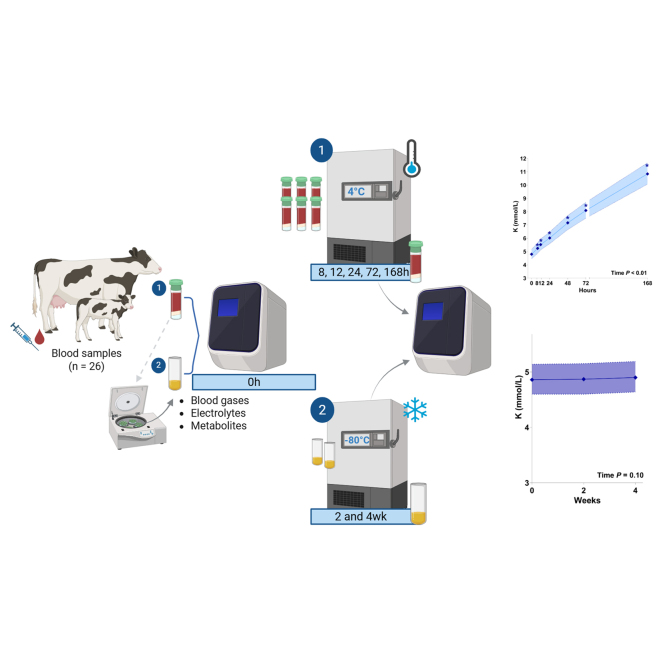
**Summary:** Blood samples (n = 182) were collected from Holstein lactating cows and preweaning heifers (n = 26) at a single time point. One whole blood sample was analyzed as the baseline, and the remaining 6 samples were stored at 4°C for analysis of blood gases, electrolytes, and metabolites at different time points. Additionally, plasma samples were analyzed as baseline, and 2 aliquots were stored at −80°C for analysis. Whole blood measurements of electrolytes, blood gases, and metabolites remained stable for up to 48 hours after collection when stored at 4°C. Potassium and lactate were less stable in whole blood; however, they showed greater stability and agreement in plasma samples. Created in BioRender.

Measuring blood gases, electrolytes, and metabolites provides relevant insights into an animal's health and clinical condition, enabling more accurate diagnoses and guiding treatment decisions (Lee et al., 2020). Additionally, it also has significant value in research. Blood metabolites are extensively used to evaluate energy metabolism, mineral balance, and metabolic diseases during a cow's productive life, offering a deeper understanding of physiological and pathological states (Neves et al., 2017). Similarly, common diseases in calves, such as diarrhea, can cause disturbances in acid–base and electrolyte balance, dehydration, and metabolic acidosis, which remains the primary cause of mortality in preweaning calves (Urie et al., 2018).

Moreover, calcium and creatinine levels have been identified as potential biomarkers for early detection of cows at risk of displaced abomasum in the early postpartum period (Song et al., 2020). The pH values can be used as a predictor of mortality in diarrheic calves (Trefz et al., 2017). Furthermore, blood results can help implement adequate fluid and electrolyte treatments, aiming to correct blood metabolite alterations (Wilms et al., 2024).

Portable blood gas analyzers (**BGA**) are available for on-farm measurements, with good correlation with reference analysis (Peiró et al., 2010). However, supplies for these devices are costly (e.g., i-STAT, $22/sample + equipment), and sample numbers on farms may not justify the use. Moreover, BGA offers tests like CO-oximetry, which can be unnecessary and add to costs. Therefore, clinicians often prefer specialized laboratories for customized analyses. In field studies, researchers typically collect blood samples for later laboratory processing. Due to the distance from farms and the number of animals, samples may be stored for varying periods, leading to delays and potential changes in parameters that can affect the results and their interpretations (Arbiol-Roca et al., 2020; Wang et al., 2025).

Bach et al. (2020) showed that total calcium concentrations in plasma and serum remained stable for up to 12 mo stored at −80°C. Whole blood samples were stored at 4°C for up to 14 d before separating plasma and serum, and this delay had no impact on the results. Similarly, Mg concentration changes in serum and plasma samples were minimal, with no diagnostic relevance up to 6 mo, and serum and plasma Ca concentrations remained stable for up to 3 mo when stored at −18°C (Valldecabres et al., 2025). There is a knowledge gap regarding how other blood parameters change with temperature and storage time, which can affect the measurements and clinical interpretation of blood gases, electrolytes, and metabolites, as well as whether measurements in plasma align with those in whole blood. Therefore, the objective of this study was to evaluate the stability over time of selected blood gas, electrolytes, and metabolite measurements of clinical interest in dairy cattle whole blood samples stored at 4°C, in addition to frozen plasma samples stored at −80°C.

All animal procedures were approved by the Institutional Animal Care and Use Committee (IACUC) at Cornell University (protocol #2025-0001). The sample size was calculated by selecting one blood electrolyte, potassium (K), based on a 5% type I error and 95% power. Assuming a standard deviation of 0.4 mmol/L and a detectable difference of 0.3 mmol/L in the electrolyte concentration in blood (Verma et al., 2019), 24 animals were required. Blood samples were collected from 26 animals, Holstein multiparous lactating dairy cows (n = 10) and Holstein preweaning dairy heifers (n = 16) at a family-owned commercial dairy farm in New York state. To account for variability and a wide range of values within the analyzed parameters, the study included sick multiparous lactating cows with a parity range of 2 to 7 lactations, average DIM of 128.5 d, and diagnoses of displaced abomasum, mastitis, or metritis. Additionally, preweaning heifers, with a mean age of 5 d (range 3 to 13 d), rectal temperature >39.5°C, and diagnosed with bovine respiratory disease or diarrhea by farm personnel at enrollment, were included. Healthy animals enrolled were preweaning heifers with no treatment records in Dairy Comp 305 (Valley Agricultural Software, Tulare, CA) and a negative respiratory scoring system (McGuirk and Peek, 2014).

Samples were collected between May and June 2025. Seven blood samples were collected simultaneously from each animal using 4-mL Vacutainer tubes containing 75 USP spray-dried lithium heparin (BD Vacutainer, Franklin Lakes, NJ). Blood samples were immediately placed in ice and transported to the laboratory for processing. The samples were analyzed using the Critical Care Blood Gas Analyzer Stat Profile Prime Plus (Nova Biomedical, Waltham, MA) for sodium (Na), potassium (K), chloride (Cl), ionized calcium (**iCa**), ionized magnesium (**iMg**), pH, bicarbonate (HCO_3_^−^), base excess (**BE**), anion gap (**AnGap**), glucose (**Glu**), lactate (**Lac**), creatinine (**Creat**), and BUN following the manufacturer's instructions. One blood sample was analyzed within 30 min after collection and used as the baseline (0 h). Immediately after analysis, the baseline sample was centrifuged at 2,700 × *g* for 15 min at 22°C, and the plasma samples were harvested into separate aliquots. No hemolysis was observed in the samples. Plasma baseline was immediately analyzed, and the 2 aliquots were stored at −80°C for analysis after 2 and 4 wk. The remaining 6 whole blood samples from each animal were stored at 4°C and analyzed after 8, 12, 24, 48, 72, and 168 h of storage. At each time point, blood samples were removed from the refrigerator and allowed to reach room temperature (22°C) for 10 min standing on a test tube rack before measurements were taken. The tubes containing the blood samples were carefully inverted approximately 6 times to homogenize the blood, and then the samples were processed with the BGA. The analyzer used an automated electronic system and liquid quality control to continuously monitor equipment and perform automatic controls to ensure reliable performance.

The effect of storage time on blood gases, electrolytes, and metabolites was analyzed separately for whole blood and plasma using linear mixed-effects models (LMM) in R (v. 4.3.1; https://www.r-project.org/), with time as a fixed effect and animal as a random effect. Compound symmetry was the covariance structure used. Model assumptions of residual normality and homoscedasticity were visually assessed. When necessary, outcome variables (individual blood parameters) were log-transformed to meet model assumptions. For whole blood analysis, K, iCa, iMg, Glu, Lac, Creat, BUN, and HCO_3_^−^ were log-transformed; for plasma, K, Cl, iCa, Glu, Lac, and BUN were log-transformed before being fitted into the model. Variables were subsequently back-transformed for reporting. Tukey's post hoc test was used for *P*-value correction for multiple pairwise LSM comparisons among time points. Results are presented as LSM and 95% CI. Significance was declared at *P* < 0.05. Additionally, the agreement between baseline measures of whole blood and plasma was assessed using Passing–Bablok regression and Bland–Altman plot analysis. Graphs were created using GraphPad Prism version 10.5 (GraphPad Software, La Jolla, CA).

Blood samples (n = 182) were collected and 13 blood parameters were evaluated on whole blood, with the mean value and range of pH: 7.38 (6.99–7.56), Na: 135.0 (124.0–142.9) mmol/L, K: 6.92 (3.57–19.63) mmol/L, Cl: 101.2 (96.1–108.0) mmol/L, iCa: 1.28 (0.90–1.43) mmol/L, iMg: 0.55 (0.37–0.82) mmol/L, Glu: 102.7 (56.0–186.0) mg/dL, Lac: 2.63 (0.30–8.90) mmol/L, BUN: 14.8 (3.0–186.0) mg/dL, Creat: 0.70 (0.00–6.80) mg/dL, BE: 5.07 (−19.70–17.90) mmol/L, HCO_3_^−^: 30.37 (10.90–41.64) mmol/L, and AnGap: 10.21 (1.40–22.50) mmol/L. In plasma, 10 parameters were evaluated, and the mean and range were pH: 7.62 (7.24–7.96), Na: 138.7 (128.2–163.0) mmol/L, K: 4.89 (3.49–5.95) mmol/L, Cl: 101.0 (93.2–111.9) mmol/L, iCa: 1.23 (0.84–1.43) mmol/L, iMg: 0.51 (0.30–0.88) mmol/L, Glu: 129.8 (66.0–220.0) mg/dL, Lac: 1.72 (0.30–3.80) mmol/L, BUN: 13.4 (1.0–158.0) mg/dL, and Creat: 0.49 (0.20–0.90) mg/dL.

Changes in whole blood over time compared with the baseline are shown in Table 1. There was no effect of time for whole blood iCa (*P* = 0.08), iMg (*P* = 0.26), and Creat (*P* = 0.84) concentrations. Measurements remained stable up to 168 h of storage at 4°C, with blood concentration increase of 0.1 (1.23–1.32) mmol/L for iCa, 0.1 (0.51–0.58) mmol/L for iMg, and 0.7 (1.43–1.87) mg/dL for Creat. There was an effect of time (*P* < 0.01) on HCO_3_^−^ concentrations. Differences were detected between the 12 and 168 h time points, but no differences were observed when comparing individual time points to the baseline. Changes over time were observed for Na, K, Cl, pH, BE, AnGap, Glu, Lac, and BUN (Table 1). The Cl concentration changed only after 168 h of storage (*P* < 0.01), with slightly lower concentrations, a difference of 0.6 (99.4–101.6) mmol/L when compared with the baseline. The AnGap concentrations also differed after 168 h (*P* < 0.01), with a greater concentration, showing a difference of 3.95 (11.89–14.96) mmol/L from the baseline. However, both parameters remained within the normal range, indicating no biological significance (Kaneko et al., 2008; Latimer et al., 2011).

**Table 1:** Least squares means (±95% CI) of blood gases, electrolytes, and metabolites in whole blood samples by time point

Item	Time point (h)							*P*-value (time)
	0	8	12	24	48	72	168	
Electrolyte								
Na (mmol/L)	136.6^a^	136.2^a^	136.1^a^	135.5^b^	134.5^b^	133.8^b^	131.7^b^	<0.01
	(135.2–138.0)	(134.8–137.6)	(134.7–137.5)	(134.1–136.9)	(133.0–135.8)	(132.5–135.3)	(130.3–133.2)	
K (mmol/L)	4.80^a^	5.24^b^	5.52^b^	6.02^b^	7.16^b^	8.09^b^	10.84^b^	<0.01
	(4.46–5.16)	(4.87–5.64)	(5.13–5.94)	(5.59–6.47)	(6.65–7.71)	(7.51–8.70)	(10.07–11.67)	
Cl (mmol/L)	101.1^a^	101.5^a^	101.2^a^	101.5^a^	101.3^a^	101.4^a^	100.5^b^	<0.01
	(100.0–102.2)	(100.4–102.6)	(100.1–102.3)	(100.4–102.6)	(100.2–102.4)	(100.3–102.5)	(99.4–101.6)	
iCa (mmol/L)	1.28	1.27	1.27	1.27	1.26	1.26	1.27	0.08
	(1.23–1.33)	(1.23–1.32)	(1.23–1.32)	(1.23–1.32)	(1.22–1.31)	(1.22–1.31)	(1.23–1.32)	
iMg (mmol/L)	0.53	0.53	0.54	0.54	0.55	0.56	0.54	0.26
	(0.50–0.56)	(0.50–0.57)	(0.51–0.57)	(0.51–0.57)	(0.52–0.58)	(0.52–0.59)	(0.51–0.58)	
Blood gas								
pH	7.42^a^	7.41^a^	7.40^a^	7.40^a^	7.36^b^	7.34^b^	7.27^b^	<0.01
	(7.38–7.46)	(7.37–7.45)	(7.36–7.44)	(7.36–7.44)	(7.32–7.40)	(7.30–7.38)	(7.23–7.31)	
HCO_3_^−^ (mmol/L)	30.83^a^	30.78^a^	30.87a,b, c,d	30.48^a^	29.55^a^	30.21^a^	29.15a,b, c,d	0.01
	(28.53–33.12)	(28.49–33.08)	(28.57–33.16)	(28.19–32.77)	(27.26–31.84)	(27.92–32.50)	(26.85–31.44)	
BE (mmol/L)	6.21^a^	5.98^a^	5.91^a^	5.44^a^	4.61^b^	4.24^b^	2.06^b^	<0.01
	(3.58–8.84)	(3.58–8.84)	(3.28–8.54)	(2.81–8.07)	(1.98–7.24)	(1.61–6.87)	(−0.58–4.69)	
Anion gap (mmol/L)	9.47	9.22	9.58	9.62	10.23	10.51	13.42	<0.01
	(7.94–11.00)	(7.69–10.75)	(8.05–11.12)	(8.08–11.15)	(8.70–11.77)	(8.98–12.04)	(11.89–14.96)	
Metabolite								
Glu (mg/dL)	106.3^a^	105.3^a^	103.6^a^	102.5^a^	96.4^b^	94.6^b^	83.4^b^	<0.01
	(95.1–118.8)	(94.3–117.7)	(92.7–115.7)	(91.7–114.6)	(86.2–107.7)	(84.7–105.8)	(74.6–93.2)	
Lac (mmol/L)	1.19^a^	1.56^b^	1.65^b^	1.85^b^	2.40^b^	3.07^b^	4.67^b^	<0.01
	(0.95–1.50)	(1.24–1.95)	(1.31–2.06)	(1.48–2.33)	(1.92–3.01)	(2.45–3.85)	(3.72–5.86)	
Creat (mg/dL)	1.57	1.62	1.56	1.58	1.62	1.53	1.64	0.84
	(1.37–1.80)	(1.41–1.85)	(1.37–1.79)	(1.38–1.81)	(1.41–1.86)	(1.33–1.76)	(1.43–1.87)	
BUN (mg/dL)	8.24^a^	8.26^a^	8.27^a^	8.27^a^	8.64^b^	8.89^b^	9.32^b^	<0.01
	(1.23–1.32)	(5.87–11.62)	(5.88–11.63)	(5.88–11.63)	(6.14–12.15)	(6.32–12.50)	(6.63–13.11)	

a,b Within a row, means with different superscripts differ at *P* < 0.05 relative to baseline.

c,d Within a row, means with different superscripts differ at *P* < 0.05 relative to different time points.

There was an effect of time on the concentrations of Na, pH, BE, and Glu (*P* < 0.01), which decreased over time. In contrast, BUN concentration increased over time (*P* < 0.01). Differences were observed after 48 h: −1.6 mmol/L (1.98–7.24) for BE, −9.9 mg/dL (86.2–107.7) for Glu, and 0.4 mg/dL (6.14–12.15) for BUN compared with baseline concentrations. The continuous metabolic activity of mature red blood cells may explain Glu concentration levels. In anaerobic metabolism, the most common process is glycolysis (Gokce et al., 2004). As a result, Glu is consumed over time, and lactic acid accumulates, which may also be associated with a decrease in blood pH. A change over time was observed for pH (*P* < 0.01) and Na (*P* < 0.01) at 24 h, with lower values of pH 7.40 (7.36–7.44) and Na 135.5 (134.1–136.9) mmol/L when compared with the baseline pH 7.42 (7.38–7.46) and Na 136.6 (135.2–138.0) mmol/L. Nevertheless, the values remained within the normal range (Kaneko et al., 2008), making them reliable for clinical purposes for up to 48 h. The pH values agreed with those reported by Gokce et al. (2004), who found that measures of pH in bovine venous blood stored at 4°C remained stable and within the normal range up to 48 h.

Concentrations of K and Lac were less stable, showing significant increases after 8 h (*P* < 0.01). At baseline, concentrations were 4.80 (4.46–5.16) mmol/L for K and 1.19 (0.95–1.50) mmol/L for Lac. After 8 h, concentrations increased to 5.24 (4.87–5.64) mmol/L and 1.56 (1.24–1.95) mmol/L, respectively. Similarly, Menta et al. (2020) reported changes in whole blood K concentrations with clinical relevance as early as 4 h after storage. The increase in K concentrations in blood at lower temperatures (4°C) is explained by the temperature-induced inhibition of the Na-K pump, causing the release of K from cells (Oddoze et al., 2012). Accordingly, the same mechanism causes lower Na concentrations. Whereas Menta et al. (2020) found a significant decrease in Na after 8 h of storage, our findings suggest that Na remains stable up to 24 h, but measurements are still within the normal range and reliable for clinical purposes for up to 48 h.

Plasma sample measurements and their concentrations over time are presented in Table 2. Plasma concentrations for Na (*P* = 0.24), K (*P* = 0.10), iMg (*P* = 0.86), and BUN (*P* = 0.45) did not change within 4 wk of storage at −80°C compared with the baseline (Table 2). There was an effect of time on plasma Lac concentrations (*P* = 0.03). However, the difference was observed between 2 and 4 wk, with no differences when comparing time points to the baseline. An effect of time was observed for Cl, iCa, pH, Glu, and Creat (*P* < 0.01). The Cl concentration was lower at 2 wk of storage, 1.6 mmol/L (98.4–101.5) difference compared with the baseline. The iCa concentrations differed by −0.06 mmol/L (1.16–1.27) at 2 wk and −0.07 mmol/L (1.15–1.26) at 4 wk, compared with the baseline 1.27 (1.21–1.32) mmol/L. However, both parameters were within the normal range. The pH values increased over time (*P* < 0.01). However, even at the baseline measurement of plasma pH, it was greater than the blood pH, falling outside the normal range, with an error greater than ±0.04 units. These findings are in agreement with another study, which showed plasma pH was approximately 0.2 units higher than whole blood (Ro et al., 2020). Indicating that plasma pH is not a reliable method for accurate measurement, at least not without a correction factor. The Glu concentration increased after 2 wk compared with the baseline (*P* < 0.01). Like pH values, Glu levels were higher at the first plasma measurement, in agreement with the literature, which indicates that whole blood Glu levels are lower due to the lower water content of erythrocytes (Sacks et al., 2002). The concentration of Creat was higher at 4 wk, 0.08 mg/dL (0.49–0.6) difference compared with baseline. Although plasma concentrations were lower than those in whole blood, the values fell within the normal range and with acceptable variation of ±0.2 mg/dL (Verma et al., 2019). Flores et al. (2020) evaluated the stability of human plasma and serum under different storage conditions. They found that plasma and serum are equivalent, and storing samples at −20°C for up to 30 d maintains Glu and Creat measurements without any clinically significant difference.

**Table 2:** Least squares means (±95% CI) of blood gases, electrolytes, and metabolites in plasma samples by time point

Item	Time point (wk)			*P-*value (time)
	0	2	4	
Electrolyte				
Na (mmol/L)	138.1 (136.1–139.9)	138.3 (136.4–140.3)	139.4 (137.5–141.4)	0.24
K (mmol/L)	4.86 (4.60–5.14)	4.87 (4.60–5.15)	4.90 (4.64–5.19)	0.10
Cl (mmol/L)	101.5 (99.8–103.1)^a^	99.9 (98.4–101.5)^b^	101.2 (99.6–102.9)^a^	<0.01
iCa (mmol/L)	1.27 (1.21–1.32)^a^	1.21 (1.16–1.27)^b^	1.20 (1.15–1.26)^b^	<0.01
iMg (mmol/L)	0.51 (0.47–0.55)	0.50 (0.47–0.54)	0.50 (0.46–0.54)	0.86
Blood gas				
pH	7.55 (7.51–7.58)^a^	7.66 (7.63–7.70)^b^	7.65 (7.62–7.69)^b^	<0.01
Metabolite				
Glu (mg/dL)	123.0 (108.0–139.0)^a^	126.0 (111.0–143.0)^b^	127.0 (112.0–144.0)^b^	<0.01
Lac (mmol/L)	1.45 (1.13–1.85)^a^	1.45 (1.13–1.85)a,b, c,d	1.50 (1.18–1.92)a,b, c,d	0.03
Creat (mg/dL)	0.48 (0.41–0.54)^a^	0.46 (0.39–0.53)^a^	0.56 (0.49–0.62)^b^	<0.01
BUN (mg/dL)	6.33 (4.22–9.50)	6.30 (4.20–9.46)	6.38 (4.25–9.58)	0.45

a,b Within a row, means with different superscripts differ at *P* < 0.05 relative to baseline.

c,d Within a row, means with different superscripts differ at *P* < 0.05 relative to different time points.

Interestingly, measurements of K and Lac levels remained stable for up to 4 wk in plasma samples, but were less stable and increased notably after 8 h of storage in whole blood (*P* < 0.01). Passing–Bablok regression and Bland–Altman plot analysis between whole blood and plasma measurements are presented in Figure 1. The Pearson correlation coefficient between whole blood and plasma concentrations for K (r = 0.99, *P* < 0.01) and Lac (r = 0.98, *P* < 0.01) indicated a strong correlation. Bland–Altman confirmed the good agreement between methods, with a mean bias and 95% limits of agreement for K and Lac of 0.07 (−0.10, 0.25) mmol/L and 0.21 (0.05, 0.48) mmol/L, respectively. Overall, plasma K and Lac levels closely reflect whole blood measurements, with no clinically significant differences, supporting their use interchangeably, especially due to their lower stability in whole blood. Plasma K was previously described as having a good agreement with whole blood samples (Trefz et al., 2018).

**Figure 1:**
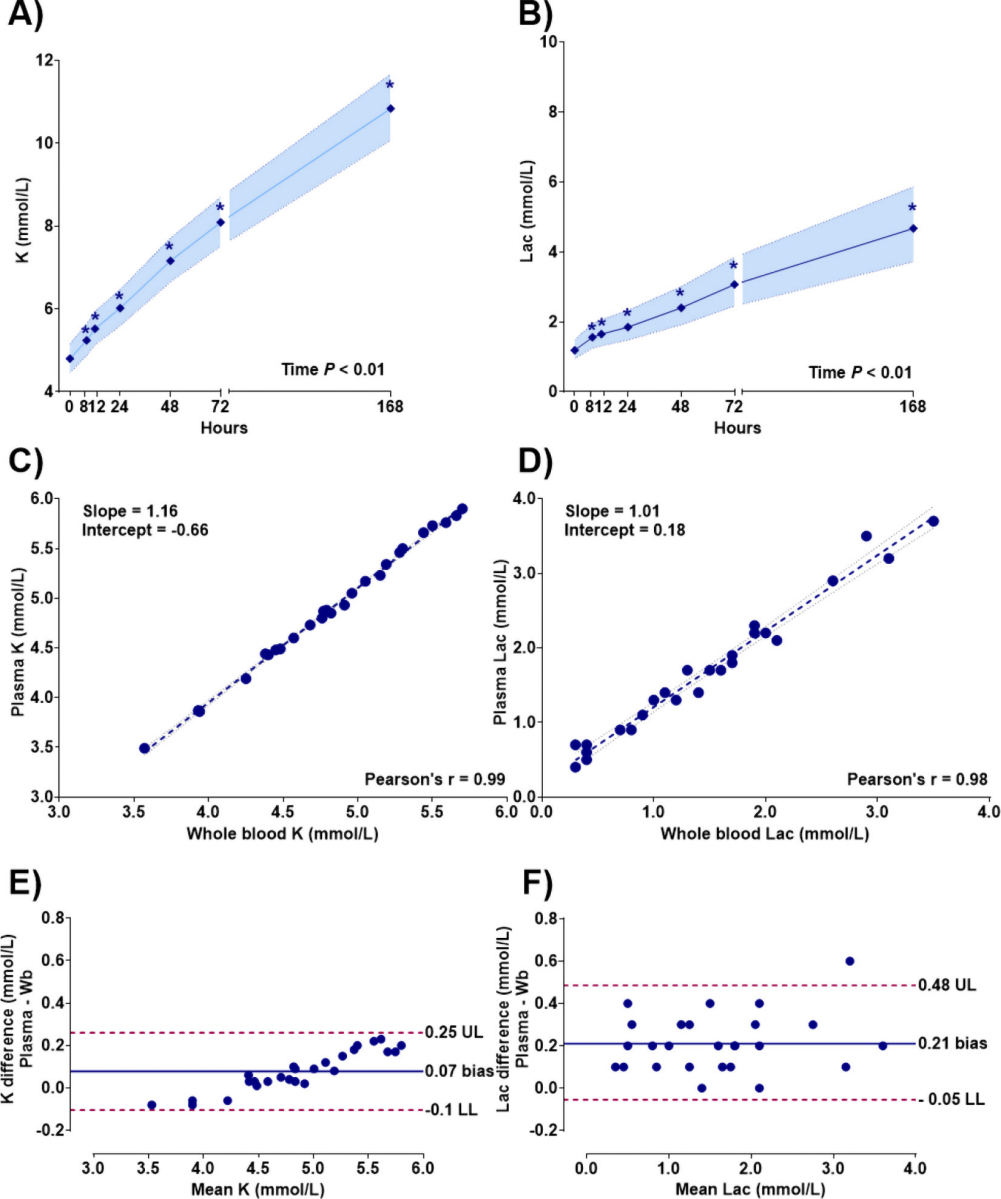
Least squares means (±95% CI) of metabolites in whole blood over time. (A) Potassium concentration over time. (B) Lactate (Lac) concentration over time. Linear regression analysis between blood metabolite concentration (mmol/L) measured in whole blood and plasma. (C) Potassium plasma concentration versus whole blood. (D) Lactate plasma concentration versus whole blood. Bland–Altman plots of the difference in metabolite concentrations. (E) Plasma K versus whole blood K. (F) Plasma Lac versus whole blood Lac. The solid horizontal line represents the mean bias; horizontal dashed lines represent the 95% CI of agreement. Wb = whole blood; UL = upper limit; LL = lower limit.

One of the study's limitations was enrolling only sick lactating cows, despite including healthy and sick calves for variability. Samples in different blood tubes may have caused variations, but this method prevents oxygen exposure and measurement interference. Additionally, plasma samples were evaluated after only 4 wk of storage, whereas longer storage is common in research. Sensitivity and specificity testing was not included, which could have offered further information for diagnostic decisions based on the storage time.

To summarize, we evaluated the stability over time of selected blood gas, electrolyte, and metabolite measurements of clinical interest in the whole blood and plasma of dairy cattle. Our findings suggest that delays in sample analysis do not clinically interfere with selected whole blood parameters if the samples are analyzed within 48 h after collection and kept refrigerated at 4°C, allowing an extended interval between collection and analysis without compromising the reliability of results.
